# Basic concepts of grain-boundary structure and phase behavior: From theory and experiments to material properties

**DOI:** 10.1557/s43577-025-01040-4

**Published:** 2026-02-23

**Authors:** Shen Dillon, Gerhard Dehm

**Affiliations:** 1 Department of Materials Science and Engineering, University of California, Irvine, Irvine, USA; 2 Max-Planck-Institut für Nachhaltige Materialien GmbH, 40237 Düsseldorf, Germany

**Keywords:** Complexion, Grain-boundary phase, Defect phase, Disconnection, Phase junction

## Abstract

**Abstract:**

Understanding and controlling structure–processing–properties–performance relationships form the central pillar of materials science and engineering. Formation of phases and evolution of material imperfections (defects) provides the two primary features of a system that enables control of these relationships. Although the impact of imperfections such as dislocations or grain boundaries on material properties has been explored quite deeply, little is known about the thermodynamic phases of the defects themselves. In recent decades, a growing appreciation for the occurrence of phase transformations of surfaces and grain boundaries has emerged. This concept of grain-boundary phase transformation and its impact on properties is at the core of this issue and introductory article. The thermodynamic fundamentals will be explained, experimental and theoretical tools to uncover grain-boundary phases and related property changes are discussed and applied to different material systems. In addition, we also want to look beyond and introduce the readers to novel findings on phase transformations of other defects, such as dislocations. In several cases, phase transformations of defects have been demonstrated to dramatically affect their properties and in turn, the overall properties of the bulk materials containing them. The additional ability to control materials properties and performance by tailoring both defect distributions and their thermodynamic phase state motivate ongoing theoretical, computational, and experimental efforts to understand and control defect phase behavior.

**Graphical abstract:**

**Figure:**
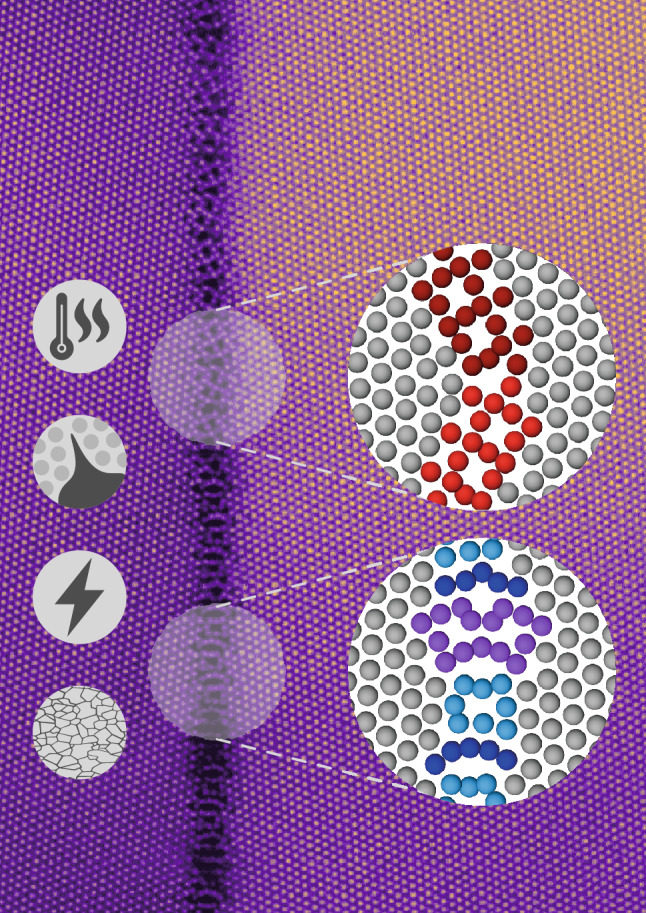
Grain boundary with two different phases. Properties like grain growth, conductivity, strength and fracture as well as thermal transport are impacted by grain boundary phases. Schematic created by Pankti Mehta (MPI SusMat) based on a TEM image of Lena Langenohl and atomistic grain boundary structures obtained by atomistic simulations by Tobias Brink (ref.16).

## Introduction

**Figure 1:**
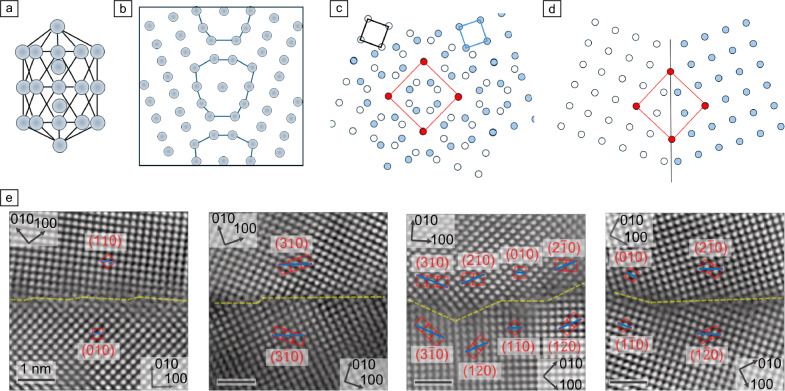
(a) One possible three-dimensional structure unit of a ∑9 ❬110❭ {221} grain boundary and (b) the corresponding two-dimensional structure (redrawn after Seki et al.^26^). (c) Coincidence site lattice (CSL) of two overlapping cubic lattices in ❬100❭ projection and (d) schematic of a symmetric ∑5 ❬100❭ {310} grain boundary. Red square indicates CSL unit cell and red circles show CSL points. A ❬100❭ tilt axis is perpendicular to the image plane. (e) Experimental high-angle annular dark-field-scanning transmission electron microscopy (HAADF-STEM) images of a ∑5 ❬100❭ tilt grain boundary with different inclinations, including symmetric {310} and {210} and asymmetric {110}//{100} segments.^27^ Taken from Zhou et al.,^27^ licensed under Creative Commons Attribution 4.0 InternatIonal License (http://creativecommons.org/licenses/by/4.0/).

Technological requirements for materials increasingly require improved performance, function in extreme environments, cost-effectiveness, and sustainable processing. A growing need, thus, exists for better understanding and exploitation of defects and their phase transitions to obtain materials with exceptional and tailor-made properties.^1–5^ As an analogue to bulk phase behavior where a face-centered cubic (fcc) and body-centered cubic (bcc) phase of the same alloy will have different physical properties, different grain-boundary phases should also exhibit distinct physical properties. Grain boundaries can be considered as being built from repeating three-dimensional (3D) structural units,^6,7^ which for simplicity are displayed often in two-dimensional (2D) projection (see **Figure** **1**a–b). The misorientation angle between two neighboring grains controls which structural unit(s) are required to merge both grains at this interface. Interestingly, not only is one configuration able to form a grain boundary of a specific crystallographic character, but several different atomic arrangements can accommodate the same misorientation. Typically, they differ in energy. Also, the number of atoms required to form the grain boundary can be different for different stable or metastable phases. This insight is key to thermodynamic considerations for the formation of grain-boundary phases, where the grain-boundary free energy, γ*,* is defined according to Frolov and Mishin^8^ as a function of:

1 $$\upgamma A{\mkern 1mu} = {\mkern 1mu} \left[ U \right]N_{1} {\mathrm{~}} - T\left[ S \right]N_{1} {\mathrm{~}} - {\mathrm{~}}\sigma _{{33}} \left[ V \right]N_{1} {\mathrm{~}} - \sigma _{{32}} \left[ {B_{2} } \right]N_{1} {\mathrm{~}} - \sigma _{{31}} \left[ {B_{1} } \right]N_{1} - \sum _{{i = 2}}^{C} \upmu _{i} \left[ {N_{i} } \right]N_{1}.$$

Here *A* is the grain-boundary area, the excess quantities are inner energy, [*U*], entropy, [*S*], volume, [*V*], excess shears in the grain-boundary plane, [*B*_2_] and [*B*_1_], the number of atoms, [*N*_i_], the other variables are temperature, *T*, excess volume, σ_33_, shear stress along the grain-boundary plane, σ_32_ and σ_31_, and µ_*i*_ the chemical potential of component *i* = *1* for a single element system or *i* = *2* for a binary alloy system. The grain-boundary structure with the lowest grain-boundary free energy γ forms the equilibrium phase. From Equation 1, it becomes clear that temperature changes, stresses, number of atoms, and/or chemical composition can induce phase transitions. The phases of grain boundaries and their transition are also often called “complexions” and complexion transition.^9^ See the next section for some remarks on the terminology. The occurrence and treatment of grain boundaries and their phases is similar to what is known for surfaces and surface reconstructions.^10,11^ Note that grain boundaries, similar to surfaces or dislocations, cannot exist alone. They always need adjacent crystal lattices. Although a single crystal has a lower total free energy than a polycrystal, this does not mean that *grain-boundary phases* can't exist. Thermodynamically they must be treated with respect to their 2D nature as already laid out by Gibbs for dividing surfaces in liquids^12^ and later for crystals by Hart^13^ and Cahn^14^ and finally the further refined treatment by Frolov and Mishin,^8^ manifested in Equation 1. Two grain-boundary phases are in equilibrium if they possess the same grain-boundary free energy. In reality, grain-boundary phases can coexist due to retarded mobility of the phase line separating both phases,^15,16^ similarly as bulk phases can coexist outside their equilibrium conditions due to sluggish phase transformation kinetics.

## A word on terminology: Grain-boundary phases versus “complexions”

Grain-boundary and line defect phases have been referred to as “complexions”^17,18^ following the introduction of the term by Tang, Carter, and Cannon two decades ago.^9,19^ The motivation for the terminology was twofold. First, defect phases only exist in the presence of the abutting bulk phase, and the term was intended to emphasize this fact. Second, the terminology intends to replace a multitude of other prior terminology describing different types of grain-boundary structures observed in various systems, such as intergranular films, amorphous intergranular phases, multilayer adsorbates, etc.^20^ In some respects, having distinct terminology provides various levels of convenience for searching, aggregating, and classifying information. There are two primary arguments against the term “complexions.” First, the terminology is not required as a grain-boundary phase (or defect phase) is well defined thermodynamically (see also the article by Frolov, Neugebauer, and Mishin, this issue^21^). Second, the terminology can contribute to confusion particularly in relation to subtleties such as first- versus second-order phase transitions and equilibrium and nonequilibrium defect phases. Although this discussion is confusing to newcomers in the field, we want to make clear that the terms should be understood interchangeably as they have been used by different authors in the literature.^22–25^ Regardless of the terminology used, the thermodynamic concept is the same. Care should also be taken to avoid confusion related to the presence of secondary bulk phases located at grain boundaries that are sometimes called grain-boundary phases—a wrong and misleading wording in the context as discussed here.

## Grain boundaries: Basic concepts and examples for single elemental metals and more complex crystal structures

Grain boundaries cover a multidimensional space, where three macroscopic degrees of freedom are required to describe the orientation of the joining grains, two macroscopic degrees of freedom describe the orientation of the grain-boundary plane with respect to those grains,^28,29^ and three microscopic degrees of freedom define the atomic shifts in *x*, *y*, and *z* directions at the grain boundary.^30^ Thus at least eight degrees of freedom need to be considered, but furthermore, the inclination of the grain boundary in a polycrystal can change (“curved” grain boundaries), faceting can occur, changes in chemical composition, changes in the number density of atoms at the grain boundary (e.g., sink for vacancies, interstitially and substitutionally occupied sites in the structural units), as well as line defects with step and/or Burgers vector (disconnections) components are additional features that need to be considered for a complete evaluation of grain boundaries and their phases. This complex situation is often simplified by using model grain boundaries, typically so-called “special” grain boundaries. “Special” grain boundaries follow a coincidence site scheme, where specific misorientation between the two grains leads to a coincidence site lattice (CSL)^31^ with a new periodicity of matching sites (Figure 1c–d). Every xth atom of grain 1 resides at the same position as an atom of grain 2. This repeat unit, *x*, is given as the sigma value of a grain boundary. For example, in a ∑5 grain boundary both lattices share the position of every fifth atom (Figure 1c–d). The common axis around which the grains are tilted/rotated needs to be identified (e.g., ∑5 ❬100❭) for a cubic material. In case of a common tilt axis contained in both grains and the grain-boundary plane, a tilt grain boundary emerges, while for a common rotation axis perpendicular to the grain boundary a twist grain boundary forms. The merging planes at the grain boundary can be the same on both sides (symmetric) or different (asymmetric), as labeled in Figure 1. A ∑5 ❬100❭ {310} grain boundary describes a symmetric grain boundary with both grains terminating with a {310} plane, while ∑5 ❬100❭ {011}//{001} describes an asymmetric grain-boundary scenario (see Figure 1d–e). Note that most real grain boundaries are mixed with tilt and twist components and asymmetric in nature.^32,33^ Investigations of special boundaries within the literature tend to be over-represented relative to their occurrence in real polycrystals owing to them being amenable to electron microscopy characterization and atomistic simulation. Nevertheless, the special grain boundaries taught the community many details on the atomistic structure–property relationships, while general grain boundaries are still largely unexplored.

The previously described complexity and the fact that grain boundaries are usually internally “buried” defects make experimental study more involved than for surfaces, which were studied by diffraction techniques such as reflection high- energy electron diffraction (RHEED) and low-energy electron diffraction (LEED) prior to the invention of atomic force microscopy (AFM) and scanning tunneling microscopy (STM) techniques. The experimental breakthroughs for grain boundaries required the advent of atomically resolved imaging using (scanning) transmission electron microscopy (S/TEM) techniques, which became available in the late 1980s (e.g., Reference 34), and reached easy-to-interpret atomic resolution high-angle annular dark-field (HAADF) STEM images with the advent of aberration correction in the early 2000s (e.g., References 35, 36). Much earlier, transitions in the morphology of grain boundaries have been noticed and studied later by *in situ* TEM as in the seminal work of Balluffi et al.^37^ on faceting transitions of grain boundaries for the system Cu-Bi.

**Figure 2:**
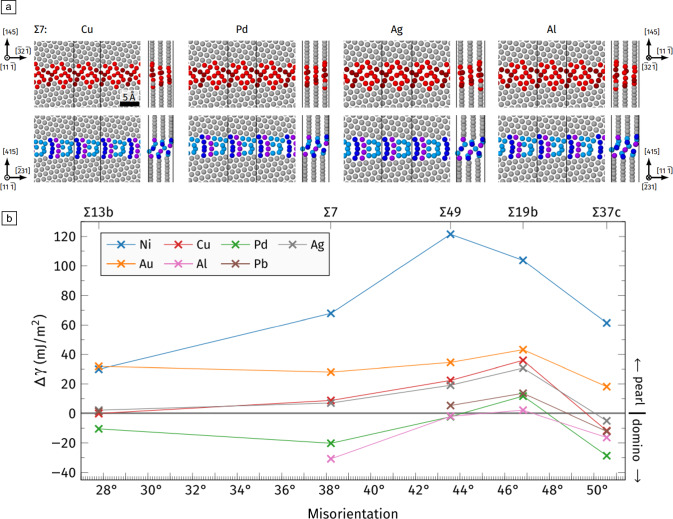
(a) Top view and cross-sectional view of symmetric ∑7 ❬111❭ tilt grain-boundary structures for different face-centered-cubic metals obtained by molecular statics simulations and (b) prediction of ground state energy difference at 0 K as a function of misorientation for two possible grain-boundary phases, domino (red structural units in [a]) and pearl (blue motifs in [a]). Figures taken from Brink et al.^43^ under the Creative Commons Attribution 4.0 International License.

Another important route to establish thermodynamic concepts for grain-boundary phases and transitions is based on atomistic simulations. Density functional theory (DFT) as well as molecular statics and molecular dynamics are the main tools used nowadays. While multiple grain-boundary structures with near degenerate energies were known to exist theoretically,^38–40^ their ability to exist in equilibrium at different conditions and the pathways for their transformation had not been appreciated in the past. A major breakthrough in the topic of grain-boundary phase transition in elemental metals was the insight that grain-boundary phase transitions may often be mediated by diffusion (i.e., transport and exchange of atoms and vacancies between the grain boundary and the surface [and/or volume]).^15^ This caused a grain-boundary phase transformation in the previously mentioned symmetric ∑5 ❬100❭{210} and {310} tilt grain boundaries. It switches at ~800 K from a “kite” to a “split kite” structure,^15^ two different structural units representing the arrangement of the grain-boundary atoms. The computational search for a possible grain-boundary phase transition was stimulated by chemical tracer diffusion of Ag along copper bicrystal boundaries indicating an abrupt change in grain-boundary diffusion at ~800 K.^41^ For ❬111❭ tilt grain boundaries in copper^42^ and later for other fcc metals,^43^ Meiners et al.^42^ and Brink et al.^43^ found that grain-boundary transitions do not necessarily require diffusion; the atomic content in the phases found for a ∑19 ❬111❭ as well as a ∑37 ❬111❭ tilt grain boundary stays constant.^16,42^ The congruent grain-boundary transition is diffusionless and caused for the Cu ∑37 ❬111❭ by entropy differences upon annealing,^16^ while stresses are required to induce a phase transformation of the Cu ∑19 ❬111❭ tilt grain boundary.^42^ Another important finding of atomistic simulations is that the structural units of different fcc elemental metals remain similar and are mainly determined by the first and second nearest-neighbor atoms and highest packing density^43^ as favored by metallic bonding. Nevertheless, different fcc metals and different types of ∑-grain boundaries show different transition temperatures or remain in single phase state^43^ (see **Figure** **2**).

In ceramics, unexpected grain-boundary phases were already discovered in the late 1970s. Lou et al.^44^ first observed intergranular films in ceramics, specifically Si_3_N_4_ containing oxide impurities. These films exhibit a constant structural thickness, typically a few nanometers, and compositions distinct from glassy phases formed at triple junctions or eutectic liquids.^45,46^ These films are known to dramatically alter ceramic processing and properties in numerous systems.^47–50^ Their impact was known to be so important that many studies have previously and sometimes still discuss the absence of intergranular films at grain boundaries as representing “clean” boundaries. Intergranular films, however, simply represent one type of grain-boundary phase observed at ceramic boundaries.^17^ Carter et al.^51^ observed the coexistence of two distinct grain-boundary phases in the same specimen of MgAl_2_O_4_ spinel ∑3 boundaries in 1987. Concurrently, Merkle and Smith observed two grain-boundary phases coexisting, separated by a disconnection-like defect (a line phase junction), at a NiO ∑5 grain boundary.^52^ The sensitivity of ceramics to impurities and variations in stoichiometry make it challenging to experimentally assess when grain-boundary phase behavior represents “intrinsic” versus chemically induced phase transitions.

## Grain boundaries: Impact of alloying and segregation

When considering how impurity, alloying, and dopant elements segregate to boundaries, it is intuitive to begin with the pure equilibrium grain-boundary structure as a template. The segregating species could then be expected to segregate to preferential substitutional or interstitial sites.^53–55^ From the point of view of the structural unit model, segregating species could introduce new structural units via their differences in size and bonding as recently observed for Ti(Fe) and Mg(Ga) grain boundaries.^56,57^ Segregation-induced changes in structural units comprising the grain boundary could cause the boundary structure to change continuously or discontinuously with changes in chemical potential. As the width of the boundary increases, as measured by structural units different from the lattice, they may become more prone to appearing disordered when imaged in a TEM, especially when observing “non-special” boundaries (i.e., boundaries with twist component, asymmetric boundaries and high-$\mathrm{\Sigma }$ boundaries). The degree of order within different types and thicknesses of intergranular films remains an open question. Nevertheless, they are more ordered than amorphous materials and less ordered than the abutting crystalline phases.^58^ Thicker disordered complexions tend to be more prevalent in ceramics, but have also been observed in metallic systems.^59–61^ It should be noted that the apparent structural, chemical, and space charge widths of boundaries can differ and are typically treated thermodynamically in context of planar excess volume, solute, or charge. Such considerations are discussed in detail in the companion manuscripts by Zhang et al.^62^ and Frolov et al.^21^

## Method developments

A significant leap forward in atomistic simulations is currently made with the development of machine learning potentials, which will permit researchers to study the thermodynamics of imperfections in crystals suffering from impurity segregation, containing alloying elements (binary and ternary systems) or expanding on more complicated crystal structures such as intermetallic materials, semiconductors, and ceramics. Machine learning potentials will provide strategies to avoid costly *ab initio* methods such as DFT calculations limited to a few hundreds of atoms.^63^ This aspect is important in order to allow large grain-boundary structural units to form as well as extended facets. The small repeat units of a few atomic layers often used even in molecular dynamics simulation can impact defect properties such as the mobility of grain-boundary phase junctions. This line defect is essential for the propagation of the equilibrium grain--boundary phase in a transition. Several approaches such as artificial neural networks trained on DFT data (e.g., References 64 and 65), or atomic cluster expansion,^66^ are used and summarized for interested readers in a recent review article by Thiemann et al.^63^

**Figure 3:**
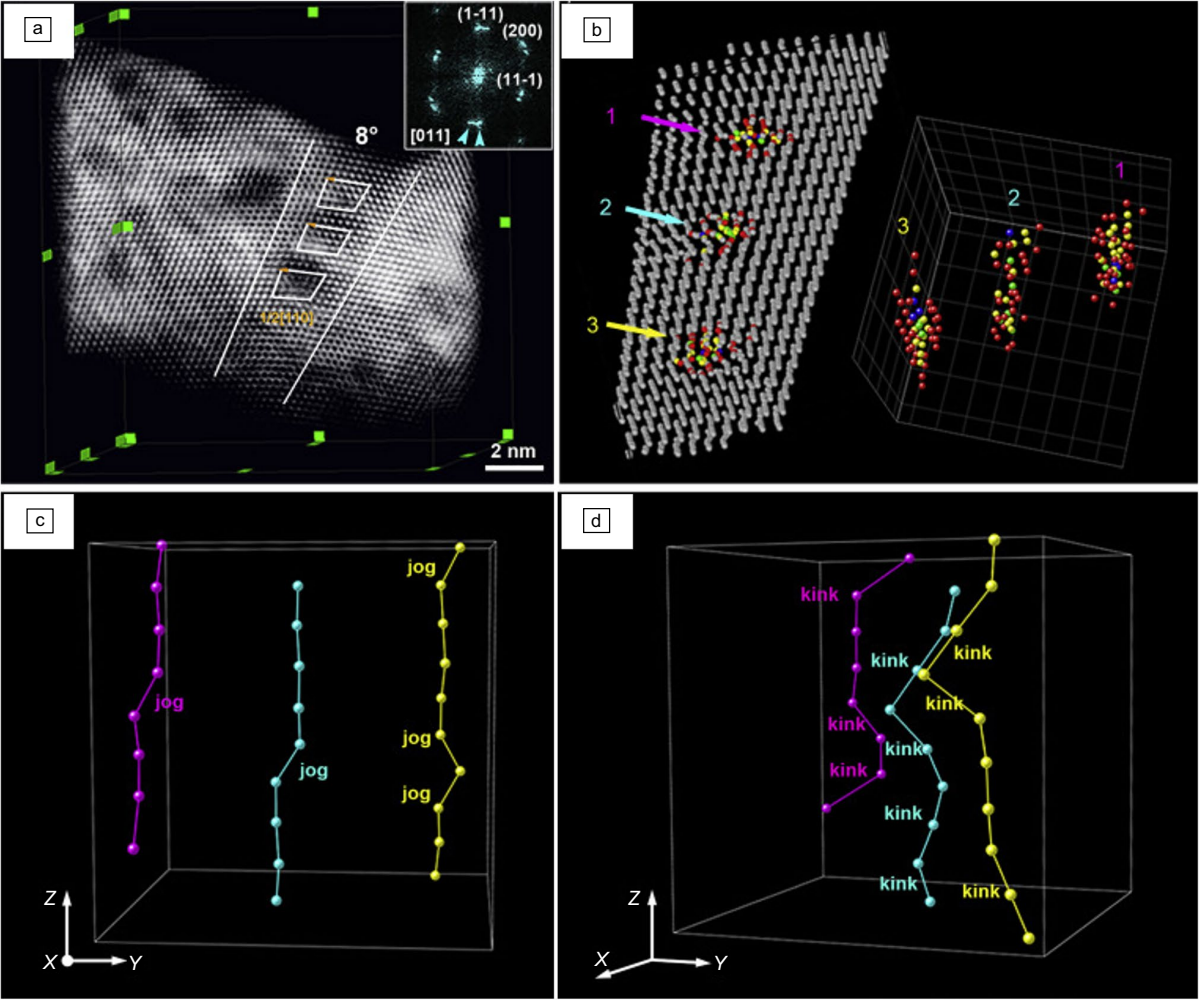
(a) Low-angle grain boundary in nanoporous Au and (b) its 3D reconstruction revealing dislocations of ½ ❬110❭. Inset in (a) reveals the power spectrum of the grain boundary. Atoms with coordination numbers smaller than 12 (face-centered cubic) are displayed for the grain boundary in color in (b). The color code corresponds to different coordination numbers. (c) and (d) present the three dislocations (1: magenta, 2: cyan, 3: yellow) imaged along different directions. Jogs and kinks are resolved along the dislocations. Reprinted with permissoin from Reference 67. © 2020 Elsevier.

Experimentally, two methods have seen tremendous improvement in recent years in obtaining 3D information of crystals and in some cases of defects. These are atom probe tomography (APT) and S/TEM tomography. For STEM and TEM tomography images are recorded at every 1 to 2° of tilt angle within a tilt range of typically maximum −75° to +75°. Higher tilt angles usually lead to mechanical shadowing of the beam by the TEM sample.^68^ This technique provides subnanometer resolutions^69^ mainly limited by the “missing wedge” (angular regime from 75° to 90°) causing inaccuracies for reconstruction in the *z*-direction. If this missing wedge can be avoided (e.g., needle or cylindrical TEM sample), and low-index zone axes are accessible, the limits can be pushed even for defects to atomic resolution in all three dimensions. This was recently demonstrated for a grain boundary in Au by Wang et al.^67^ They mapped the atomic structure of the grain boundary by HAADF-STEM and benefited from low-index zone axes images recorded at ❬100❭ and ❬110❭. The 3D reconstruction provided the transformation matrix between both grains, the 3D structural units of the grain boundary forming a decahedron configuration, the reduced coordination number of grain-boundary atoms with 9.5 compared to 12 for the bulk, and information on grain-boundary dislocation types and line directions^67^ (see **Figure** **3)**.

**Figure 4:**
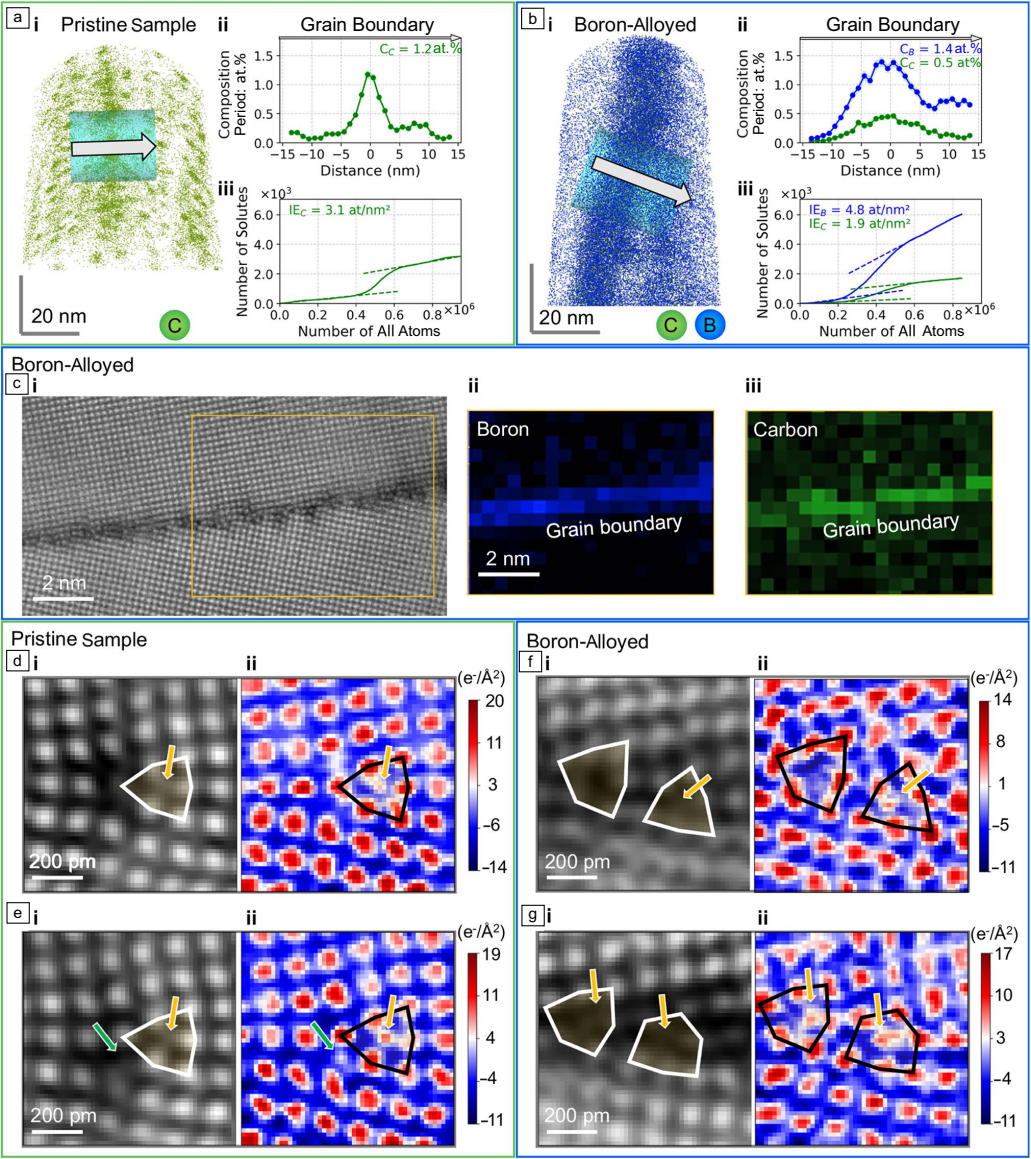
A Σ13 [001] grain boundary in pristine Fe and boron-alloyed Fe. (a) Atom probe tomography (APT) map displaying carbon (green) atoms in the pristine sample (i), corresponding line scan across grain boundary (ii) and the ladder diagram to extract the grain-boundary excess of carbon at the pristine sample. (b) Shows the boron distribution in blue (i), and compositional profile (ii) for carbon (green) and boron (blue), and the corresponding ladder diagrams (iii) with the calculated boron and carbon excess at the grain boundary. (c) Scanning transmission electron microscopy (STEM) image of the grain-boundary structure (i) and corresponding electron energy-loss spectroscopy maps for (ii) boron and (iii) carbon, revealing their presence at the grain boundary in accordance with the APT results of (b). (d–g) Four-dimensional STEM differential phase contrast imaging with (i) reconstructed dark-field and (ii) charge density maps. (d, e) Reveal carbon atomic columns at interstitial sites of the grain-boundary structural unit as indicated by orange and green arrows. (f, g) Grain-boundary structure and light element atomic positions of boron and/or carbon for the boron-alloyed sample. Figure from Reference 73 reprinted under Creative Commons Attribution 4.0 International License (http://creativecommons.org/licenses/by/4.0/).

APT is a 3D characterization technique achieving near atomic resolution.^70^ The basic principle is field evaporation of surface atoms from a needle-shaped sample with a tip radius below 100 nm exposed to a static electric field. Field evaporation is accomplished by a short electric field or laser pulses leading to sequential extraction of atoms/ions. Each ion is accelerated by the electric field toward the position sensitive detector and its mass-to-charge ratio is determined by time-of-flight spectroscopy.^71^ This procedure allows for a 3D reconstruction of the specimen with a resolution of ca. 0.2 nm along the needle-shaped sample, while a lateral resolution of ≤1 nm is achieved. This resolution can degrade at defects such as grain boundaries.^72^ The depth resolution is high as the atoms/ions are extracted layer by layer; however, the lateral position is degraded by unknown surface motion of the atoms/ions prior to their detachment from the needle apex. The stronghold of this technique is that every ion detected (detection efficiency ≤0.8) can be analyzed with respect to its chemical nature and permits observation of segregation (patterns) at defects (see **Figure** **4)**,^73^ even at low concentration levels of impurities of a few atomic parts per million.

Correlative TEM-APT requires at the moment two instruments where the needle-shaped sample is first analyzed by S/TEM techniques to determine the crystallographic details and atomic structure of imperfections such as grain boundaries and dislocations, while in a second step the sample is field evaporated in the APT. S/TEM offers a multitude of different techniques in addition to the mainly used atomic number contrast imaging by HAADF-STEM (called “Z-contrast imaging”). This includes four-dimensional (4D) data sets, where at every image position (pixel) in addition to the image intensity diffraction information is recorded, or the center of mass (COM) of the transmitted beam tracked.^74^ While the first provides access to virtual bright- and dark-field images allowing to identify Burgers vectors of dislocations,^75^ or in case of a precession of the beam can enable electron diffraction tomography,^76^ the COM technique visualizes and measures electric fields and charges in the thin specimen.^77^ Figure 4 provides a recent example where the chemical composition of the grain boundary in Fe was analyzed with respect to B and C grain-boundary segregation, whereas 4D S/TEM images provide information on the atomic structure of the grain boundary and location of B and C atoms.

## Properties and grain-boundary phases

The ability to control material properties not only through their behavior in the bulk phase, but also through their defect phase(s), opens up new avenues for material development. This has motivated research focused on understanding how defect phase behavior influences a broad variety of phenomena, including ion transport for batteries^78–81^ and fuel cells,^82^ development of ultrahard materials,^83,84^ stability under particle irradiation,^85,86^ thermoelectric performance,^87–90^ mechanical strengthening and toughening,^91–97^ geological evolution,^98,99^ the behavior of metal-oxide phase boundaries,^100,101^ or the ferroelectric response of grain boundaries.^102^ There has been considerable interest in understanding how grain-boundary phase transitions impact microstructural evolution during processes like grain growth,^17,103^ sintering,^44,104^ and creep,^105^ influence the grain-boundary texture,^106^ and thus impact collective physical properties.^97^ The multiscale nature of grain-boundary phase behavior and their impact on macroscopic properties make unambiguously establishing such correlations challenging. These observations, nevertheless, have motivated significant recent interest related to understanding, predicting, characterizing, and controlling defect phase behavior. The collection of previously cited articles and this issue reviews recent contributions to understanding defect phase behavior and its associated impact on materials processing, properties, and performance.

## In this issue

This compendium builds on past reviews of the subject^22,24,107–121^ for utilizing defect phase behavior to engineer novel material properties and to understand fundamental aspects of defect dynamics. The contributions emphasize the current state of the art in understanding defect thermodynamics and phase behavior, grain-boundary structure and chemistry in relation to their phase behavior, their impact on grain-boundary motion and transport, their influence on functional properties of materials, and the structure and properties of defect phases beyond interfaces. Here, we provide a brief summary of the most important content of the following articles in order to spark readers' interest in further information, and on suggested future research directions.

### Thermodynamics of grain-boundary phases

Classical thermodynamics provides the basis for understanding defect phase equilibrium, defining anticipated trends in the stability of equilibrium, and predicting the types of atomic, chemical, and physical interactions that drive phase behavior. Frolov and co-workers^21^ provide a historical perspective for the development of thermodynamics related to phase equilibria at 2D and 1D defects. The general nature of Gibbs’ treatment of interfacial thermodynamics lends itself directly to encompassing grain-boundary phase transformations.^122^ The article elegantly highlights the analogy between phase behavior at each dimension and the analogy between the Gibbs-Duhem, interfacial adsorption, and line defect adsorption Equations.^21^ Understanding of surficial phase transformations in single and multicomponent systems substantially predated analogous work at solid–solid interfaces.^123^ These developments are beyond the scope of this series of articles and have been reviewed elsewhere. Early contributions by Hart,^13^ Cahn,^14^ Rottman,^124,125^ and others accounted for some thermodynamic factors specific to crystalline interfaces such as symmetry, additional degrees of freedom, and elastic stress or strain. The recent development of grand canonical atomistic approaches^126,127^ have demonstrated the ability to recover experimentally observed grain-boundary structures in detail. These methods have been powerful in directly predicting grain-boundary structures.^16,56,128^ Large-scale calculations also reveal important aspects of the differences in grain-boundary volume between different phases, the strain that occurs during a transformation, and the line defects that accommodate this strain.^129,130^ These details provide a basis for predicting the thermodynamic barriers associated with transformations between phases in context of classical nucleation theory.^130^ These transitions will influence hysteresis that has been observed experimentally in various systems.^131–133^ Large-scale atomistic studies have also provided insights into the nature of microstates representing the same grain-boundary phase versus thermodynamically distinct macrostates representing different grain-boundary phases.

### Probing grain-boundary structure, chemistry, and transitions

A key challenge in understanding grain-boundary phase behavior, and its influence on material properties, is the need to characterize the structural, chemical, and field gradients that define a given phase. Zhang et al.^62^ elaborate in their article how grain-boundary transitions can be uncovered. This starts with selection of grain boundaries from materials using electron backscatter diffraction (EBSD) scanning electron microscopy (SEM) to identify the misorientation and possible joint zone axis of the adjacent grains for subsequent high-resolution S/TEM characterization. Abrupt grain-boundary property changes, such as grain-boundary failure,^93,134,135^ abnormal grain growth,^17,136^ discontinuities in electrical^137,138^ or thermal properties^139,140^ are often indicators of possible grain-boundary phase transitions as exemplified with several examples in their overview. Topological changes of the grain-boundary (facet formation),^37,73,141–143^ premelting and intergranular films^17,134,144–147^ or changes in atomic and chemical structure^56,57,148,149^ require high-resolution characterization techniques for their observation. Electron microscopy, APT, and atomistic simulations provide the most direct means of probing grain-boundary phase behavior in relation to structure and chemistry. However, each method faces significant practical hurdles, particularly in determining grain-boundary structure and chemistry in full three dimensions and under *in situ* conditions where phase transitions occur. Adding to the complexity, classification schemes for grain-boundary structure and chemistry are diverse and often intricate, drawing on structural unit models, disconnection models, and structural disorder classifications. The article by Zhang et al.^62^ offers a detailed overview and practical guide for characterizing and classifying grain-boundary structure, chemistry, and phase.

### Grain-boundary mobility and grain growth

The article by Marder et al.^150^ in this issue discusses how, contrary to traditional solute drag effects, certain dopants and alloying elements in specific metal and ceramic systems have been shown to enhance grain-boundary mobility. The phenomenon has been correlated with apparent complexion transitions in several systems. Here, apparent is denoted because it is challenging to assess when an experimentally observed grain boundary is in its equilibrium state. Indeed, the occurrence of abnormal grain growth, which is associated with the coexistence of multiple complexions, requires that some of the boundaries be metastable in order for a growth advantage to persist. These general phenomena have motivated several efforts to understand how grain-boundary phases influence their mobility and overall grain growth evolution.

Grain-boundary migration occurs primarily via the motion of disconnections, grain-boundary line defects with step and/or Burgers vector character.^151–154^ The step character of disconnections mediates grain-boundary migration. The Burgers vector character of disconnections requires that they climb and/or glide inducing grain-boundary deformation. Their motion couples to stress fields and also generates stress fields.^155^ The effects of shear coupling to grain-boundary motion are well documented in experiments and simulations.^156–158^ Since grain growth does not preserve volume some degree of point defect flux is required between sinks (i.e., triple junctions, disconnections that climb, and/or dislocations). Shear coupling along with required conservation of Burgers vector during triple junction reactions drives a so-called “network effect” where the motion of adjacent boundaries may not occur independently.^159^ These provide an explanation for the breakdown in the classically anticipated relationship between grain-boundary curvature and grain-boundary mobility predicted by traditional formulations of steady-state grain growth.^160^

Numerous questions remain with regard to how grain-boundary complexions impact grain growth. Disconnection theory provides a useful framework for understanding shear coupling and grain-boundary migration, but the influence of different grain-boundary phases on these mechanisms is unclear. It has been shown that a grain-boundary phase transformation can, for example, change the sign of shear coupling.^161^ The nature of disconnection reactions at triple junctions may also be significantly modified by grain-boundary and/or triple junction phase transformations. Naively, one could anticipate that enhancing the grain-boundary mechanical compliance, for example, by forming a structurally disordered intergranular film, could suppress back stresses that can otherwise evolve from shear coupling.

### Grain boundaries' defect phases and their impact on functional properties

Grain boundaries are often the most important imperfections in materials for functional applications. Typically, the average grain-boundary behavior is considered when efficiencies or figure of merits for applications are discussed. However, it has recently been recognized that the individual behavior of grain boundaries is as diverse as the multitude of grain boundaries that exist, opening up new strategies for improving overall properties by adjusting grain-boundary types and their chemical composition. This is the central topic of the article by Cojocaru-Mirédin et al. in this issue^162^ with a focus on energy-harvesting materials. They provide a comprehensive overview on the interplay between functional properties and grain-boundary structure as well as chemical composition. One of the most studied materials is polycrystalline Si, a key material for photovoltaic applications. Correlative electron-beam-induced current (EBIC) SEM, EBSD-SEM, atomic resolved STEM, and APT measurements revealed huge differences in electrical conductivity for different grain boundaries. Symmetric ∑3 (111) grain boundaries behave similar to the surrounding bulk, while the incoherent ∑3 (211) grain boundaries show strong losses of charge carriers by recombination, although the misorientation between the grains is the same as for the ∑3 (111), just the inclination is different. The atomic structure, local strains and stresses at facet junctions of the ∑3 (211) grain boundary as well as segregated trace impurities such as C and O cause the detrimental effect.^163–165^ The type of grain boundary in Si is also modifying thermal conductivity as revealed by recent spatially resolved frequency-domain thermoreflectance measurements.^166^ This is important for thermoelectric applications, where a balance of high electrical conductivity and low thermal conductivity is required to optimize performance. Grain boundaries appear to be a promising approach, and the field of research is currently benefiting from a better understanding of the relationships between grain boundaries and properties as reported in several publications.^87,89,90^ Similarly, grain boundaries are also key for Li and Na solid-state batteries. Unfortunately, they suffer radiation damage when exposed to electron beams making it almost impossible to achieve atomically resolved S/TEM imaging of the original state. Low-dose S/TEM at cryogenic temperature could offer new possibilities, but so far APT is a very successful method to determine, for example, Li excess and possible 2D grain-boundary phases (intergranular films). The article by Cojocaru-Mirédin et al.^162^ covers more material systems than given in this brief summary and also reflects on the impact of electrical fields on ionic conductivity in connection with grain boundaries.

### Defect phases beyond grain boundaries

This issue deals mainly with the defect phase framework for grain boundaries, but it can of course be extended to all types of defects in materials. This includes 0D defects such as vacancies, but also 1D defects such as dislocations. Some basic thermodynamic concepts are derived in the accompanying article by Frolov et al.,^21^ while a detailed presentation of the experimental findings on dislocation defect phases in alloys and ordered intermetallic materials is summarized by Korte-Kerzel et al.^167^ An early work in this direction is Kirchheim’s defectant theory,^168^ developed to understand hydrogen segregation to dislocation cores in metals. The decrease in formation energy of a dislocation (γ) in the matrix is given by the excess (Γ) and the chemical potential (μ) of the segregant

2 $$d\upgamma \,=\,\Gamma d\upmu.$$

Experimentally, it was found that hydrogen reduces the dislocation nucleation barrier in several material systems,^169,170^ including Pd-H as proven by nanoindentation.^168^ A chemically triggered phase transformation of a dislocation requires a distinct change in dislocation core structure, which needs to be proven by experiment and/or simulation. Alternatively, temperature, pressure, and, in the case of ionic or magnetic materials, electric or magnetic fields can cause phase transitions. APT measurements highlight several examples in material systems, where experimental indications of 1D defect phases are found. Examples are Fe-Mn^18^ and Pt-Au.^171^ Atomistic simulations predict structural transitions at and in dislocations outside the limits of bulk phase diagrams, as for example hydride formation at a dislocation core in Ni-H,^172,173^ or stable L_1o_ and metastable B_2_ phase coexistence at dislocation cores in FeNi,^174,175^ providing two examples among several. A detailed review on dislocations and their defect phases in bulk Laves phases and selected intermetallic materials rounds off the current understanding of 1D defect phases. While the focus is laid on mechanical properties, their influence can also extend beyond to functional properties. The authors illustrate this with examples such as the change in phonon scattering in thermoelectric materials caused by dislocations.^176^ The perspective of shaping materials design by 0, 1, and 2D defect phases leads to a number of open questions for future research. “How can we obtain defect structures by synthesis and processing according to defect phase diagrams, and what is the tradeoff between defect phases and their properties?” These are among the pressing questions raised by Korte-Kerzel et al.^167^

## Outlook and future opportunities

This article is intended to provide the nonexpert with the most important concepts concerning grain-boundary phases, sometimes also called complexions in the literature. This includes in a nutshell the underlying thermodynamic concept, as well as experimental and computational techniques to find and explore grain-boundary phases. Examples of grain-boundary phases have been resolved nowadays in a number of materials, including elemental metals, alloys, and ceramics. Some basic building principles are resolved for metals, where dense packing and nearest-neighbor bonds control the grain-boundary structure to a large extent. However, a change in the number of atoms or vacancies contained in the grain boundary (e.g., by diffusion in an open system) can stimulate new grain-boundary phases. Impurities or alloying additions can also trigger grain-boundary phase transition(s). Note that different grain-boundary phases possess different grain-boundary properties (e.g., excess volume). Segregants can change the grain boundary’s atomic structure compared to the pure grain boundary or incorporate the segregant at substitutional or interstitial sites with respect to the grain boundary’s structure units. The large number of degrees of freedom for grain boundaries (misorientation, grain-boundary inclination, atomic coordination, chemistry [segregation], local charges and magnetic moments of grain-boundary atoms/ions) make it difficult to harvest the property changes in a tailored way and need further research. Similarly, the fact that other defects, especially line defects, can undergo phase transitions requires further studies to explore this fascinating field of defect phases and their impact on material properties. Several property impacts by defect phases are described in the subsequent articles. Here, we would like to take the opportunity to highlight some selected research opportunities at the end of this article.

## Grain-boundary line defects, stability, and kinetics of grain-boundary phases

In several works,^16,130,177^ the importance of the phase junction line, a disconnection type line defect, separating two grain-boundary phases have been highlighted. For nucleation theory governing grain-boundary phase transitions, the grain-boundary phase junction line introduces the energy penalty giving rise to an activation energy for nucleation of a new grain-boundary phase. They also control the rates of equilibration in the system and give rise to the grain-boundary analogs of transition-time–temperature diagrams.^132,178^ So far very little is known by experiments on the nucleation and mobility of grain-boundary phase junction lines. Their impact on grain growth^133^ is still in its infancy and currently mainly explored by atomistic simulations. In addition to the phase junctions, grain-boundary dislocations (disconnections) are present in materials as shown in many studies (e.g., References 16 and 179). They are important for grain-boundary motion, especially under shear,^152,155,156,180^ but may be different for different grain-boundary phases^181^ or move in opposite directions under shear stress.^161^ The interplay of the grain-boundary phase junction line with grain-boundary dislocations (disconnections) on the kinetics of grain-boundary phase formation as well as mobility of grain-boundary phases under external stimuli (stress, electric fields, magnetic fields, …) is still poorly understood. It currently attracts new research attention, for example, concerning grain growth,^155,160,179,182,183^ as well as sintering and creep.^184,185^

## Tuning microstructure and response through defect phases

The importance of defects in affecting materials response often relates to their distributions and interactions. For example, the traditional concept of grain-boundary engineering involves controlling the grain-boundary character distribution to obtain higher concentrations of more favorable interfaces.^186,187^ In plasticity, control over the degree of strain localization versus homogenous deformation depends on the evolution of the dislocation structure and strongly influences deformation and failure.^188^ An outstanding challenge for exploiting defect phase behavior for materials engineering involves understanding how defect phases influence their distribution. For example, Al_2_O_3_ samples of the same chemistry but having distinct predominant grain-boundary complexions have been shown to exhibit very different grain-boundary character distributions.^189^ Dislocations can, in principle, undergo phase transitions,^18,175,190^ which could influence mechanical response. For example, it has been shown that segregation at dislocations can cause their multiplication in systems prone to liquid-metal embrittlement^191^ or hydrogen embrittlement, (e.g., References 169 and 192). In this case, it could be argued that the segregated dislocations have lower energy and different mobility that promotes embrittlement. While this does not necessarily imply complexion transitions occur in those defects, it is suggestive of how dislocation phase transformations could strongly influence materials’ physical properties.

In summary, it is worthwhile to reexamine property changes of materials while accounting for the existence of defect phases, to further deepen our understanding of the relationships between structure, processing, properties, and performance.
